# Non-invasive Mapping of Face Processing by Navigated Transcranial Magnetic Stimulation

**DOI:** 10.3389/fnhum.2017.00004

**Published:** 2017-01-23

**Authors:** Stefanie Maurer, Katrin Giglhuber, Nico Sollmann, Anna Kelm, Sebastian Ille, Theresa Hauck, Noriko Tanigawa, Florian Ringel, Tobias Boeckh-Behrens, Bernhard Meyer, Sandro M. Krieg

**Affiliations:** ^1^Department of Neurosurgery, Klinikum Rechts der Isar, Technische Universität MünchenMunich, Germany; ^2^Faculty of Linguistics, Philology, and Phonetics, University of OxfordOxford, UK; ^3^Section of Neuroradiology, Department of Radiology, Klinikum Rechts der Isar, Technische Universität MünchenMunich, Germany

**Keywords:** brain mapping, repetitive navigated transcranial magnetic stimulation, face processing, neuropsychology, preoperative mapping

## Abstract

**Background:** Besides motor and language function, tumor resections within the frontal and parietal lobe have also been reported to cause neuropsychological impairment like prosopagnosia.

**Objective:** Since non-navigated transcranial magnetic stimulation (TMS) has previously been used to map neuropsychological cortical function, this study aims to evaluate the feasibility and spatial discrimination of repetitive navigated TMS (rTMS) mapping for detection of face processing impairment in healthy volunteers. The study was also designed to establish this examination for preoperative mapping in brain tumor patients.

**Methods:** Twenty healthy and purely right-handed volunteers (11 female, 9 male) underwent rTMS mapping for cortical face processing function using 5 Hz/10 pulses. Both hemispheres were investigated randomly with an interval of 2 weeks between mapping sessions. Fifty-two predetermined cortical spots of the whole hemispheres were mapped after baseline measurement. The task consisted of 80 portraits of popular persons, which had to be named while rTMS was applied.

**Results:** In 80% of all subjects rTMS elicited naming errors in the right middle middle frontal gyrus (mMFG). Concerning anomia errors, the highest error rate (35%) was achieved in the bilateral triangular inferior frontal gyrus (trIFG). With regard to similarly or wrongly named persons, we observed 10% error rates mainly in the bilateral frontal lobes.

**Conclusion:** It seems feasible to map the cortical face processing function and to generate face processing impairment via rTMS. The observed localizations are well in accordance with the contemporary literature, and the mapping did not interfere with rTMS-induced language impairment. The clinical usefulness of preoperative mapping has to be evaluated subsequently.

## Introduction

Although complete resection of brain tumors leads to a superior oncological outcome, any neurological deficit must be avoided in order to provide the best quality of life for the patients (Stummer et al., 2008; Capelle et al., 2013). Thus, neurosurgeons strongly focus on the preservation of postoperative motor or language function and have developed several techniques to achieve this aim (Sanai et al., 2008; De Benedictis et al., 2010; Szélenyi et al., 2010; Fernández Coello et al., 2013). Yet, besides motor and language deterioration, tumor resections within the frontal and parietal lobe were also reported to cause a significant number of patients with neuropsychological impairment (Sanai et al., 2012). However, since preoperative mapping of cortical motor function was recently shown to actually improve patient outcome as well as the extent of resection, non-invasive preoperative mapping of neuropsychological functions seems a highly reasonable approach (Krieg et al., 2014a). Since the accuracy of functional magnetic resonance imaging (fMRI) suffers severely in the vicinity of tumoral lesions, another technique seems promising: transcranial magnetic stimulation (TMS). TMS has gained broader acceptance among neurosurgeons since it was fused with neuronavigation as navigated TMS (nTMS). In recent years, several studies have been published on the use of nTMS for preoperative mapping of motor and language function and showed a good correlation with intraoperative direct cortical stimulation (DCS) (Picht et al., 2009, 2012, 2013; Tarapore et al., 2012, 2013; Krieg et al., 2012b, 2014b; Sollmann et al., 2015).

There were many studies published on the neuropsychological impairment of facial processing, so-called prosopagnosia (Sergent and Signoret, 1992; DeGutis et al., 2007; Sorger et al., 2007). In general, facial processing involves a lot of different, not only cortically located subfunctions. For instance, sensory components like the somatosensory cortex, visual pathways like the occipital face area (OFA) or the fusiform face area (FFA), memory-associated structures like the hippocampus, or emotional aspects, like those processed in the amygdala, have been associated with facial processing (Atkinson and Adolphs, 2011). Furthermore, researchers were already able to detect anatomical locations for prosopagnosia in brain-damaged patients (Sergent and Villemure, 1989; Young et al., 1993; Barton, 2014).

Another important item is the differentiation between language-related errors including semantic retrieval concerning facial processing and errors generated without just involving important language pathways.

This study evaluates the feasibility and spatial discrimination of repetitive nTMS (rTMS) mapping for detection of cortical face processing areas in a cohort of healthy volunteers (Van Honk and Schutter, 2004; Pitcher et al., 2007; Atkinson and Adolphs, 2011). In this regard it has already been shown that rTMS seems feasible for precisely mapping cortical calculation function in healthy subjects (Maurer et al., 2016). Therefore, the current study focuses on three issues:

1. The feasibility of locating facial processing via rTMS per se.
2. Exploring whether differences in neuronal processing between the two hemispheres and the different stimulation spots can be detected as a measure for spatial resolution.
3. Evaluating whether our findings for cortical localization of face processing match with those of the current literature.

Hence, in this study, we provide the first steps of establishing this new technique for neurosurgeons and neuroscientists.

## Methods

### Study subjects

Twenty healthy volunteers who suffered from no cerebral pathology were enrolled (Table 1). No volunteer was taking any kind of medication. Inclusion criteria were German as mother tongue, right-handedness (Edinburgh handedness test), and age above 18 years. Exclusion criteria were general TMS and magnetic resonance imaging (MRI) contraindications (Rossi et al., 2009).

**Table 1:** **Cohort characteristics**.

**Subject No**.	**Gender**	**Age (years)**	**Correct baseline pictures**		**Pain (VAS) convexity**		**Pain (VAS) temporal**		**rMT (% output)**	
			**Left**	**Right**	**Left**	**Right**	**Left**	**Right**	**Left**	**Right**
1	F	23	25	45	2	2	5	6	28	25
2	M	25	48	57	2	3	6	6	32	39
3	M	29	55	61	2	1	6	5	37	29
4	M	25	50	56	1	1	4	7	29	25
5	F	23	46	54	0	2	4	5	27	32
6	M	25	57	62	1	1	2	2	29	28
7	F	24	34	35	2	2	4	4	35	40
8	M	21	31	45	0	1	5	3	35	31
9	M	26	38	40	5	7	6	8	37	39
10	F	23	22	26	4	1	7	5	42	33
11	F	24	33	26	4	5	7	6	38	41
12	F	23	30	27	0	2	1	6	27	27
13	F	23	46	34	2	2	3	3	40	33
14	M	26	38	34	5	4	6	7	40	33
15	F	26	29	27	1	1	3	3	39	35
16	F	24	43	41	5	5	5	7	30	29
17	M	24	20	19	4	4	7	6	30	29
18	F	23	42	33	1	2	4	3	37	32
19	M	27	58	51	2	1	3	2	35	29
20	F	27	58	54	6	4	8	5	41	32
Median	–	25	40	40.5	2	2	4.5	5	35	32
95% CI	–	24–25	35–46	35–48	1.7–3.3	1.7–3.4	4.0–5.7	4.1–5.8	32–37	30–34
P	–	–	0.694		0.975		0.992		0.997	

*This table gives an overview of the characteristics of all enrolled volunteers, including correctly named baseline pictures, pain during stimulation, and rMT. Abbreviations: F, female; M, male; rMT, resting motor threshold; VAS, visual analog scale; CI, confidence interval*.

### Study design

All volunteers underwent two rTMS mapping sessions. Both hemispheres were investigated randomly with an interval of 13–16 days between the mappings of each hemisphere. All mappings were conducted by the first author.

### Ethics

Written informed consent was provided by all volunteers prior to rTMS. Approval was obtained by the local ethics committee of our university (Ethics Committee Registration Number 5811/13) and in accordance with the Declaration of Helsinki.

### MRI acquisition

Prior to the first rTMS mapping, all subjects underwent MRI. This was performed on a 3 Tesla scanner in combination with an 8-channel phased array head coil (Achieva 3 T, Philips Medical Systems, the Netherlands B.V.). For anatomical co-registration, our scanning protocol consisted of a three-dimensional gradient echo sequence (TR/TE 9/4 ms, 1 mm^3^ isovoxel covering the whole head, 6 min 58 s acquisition time) without intravenous contrast administration. Afterwards, the 3D dataset was transmitted to the rTMS system by using the DICOM standard.

### rTMS mapping

#### Experimental setup

This setup was applied identically for all participants: rTMS mapping was performed two times using the Nexstim eXimia NBS system version 4.3 and a NexSpeech® module (Nexstim Plc., Helsinki, Finland) via 3D T1-weighted MRI (please see “Supplemental data”).

#### Facial processing task

Our data set consisted of 80 portraits/photos of popular persons in culture, entertainment, sports, and politics and was compiled in a way that volunteers between the age of 20–30 years were able to recognize and name them. The photos were selected by the first author prior to the first mapping out of the Internet or obtained from existing databases. The 80 pictures were presented in a randomized way on a 15-inch screen 20 inches in front of the volunteer. Participant answers had to be given in German. A baseline test was performed by every volunteer prior to every rTMS mapping. All falsely named, misnamed, or wrongly pronounced pictures out of the 80 items were counted and excluded from the consecutive rTMS sequence, as outlined in Table 1. (Please see “Supplemental data”).

#### Facial processing mapping procedure

Each picture was shown on a screen for 700 ms with a fixed inter-picture interval (IPI) of 3 s and 0 ms picture-to-trigger interval (PTI) (Baptiste and Fehlings, 2006). The PTI is defined as the time from displaying the picture on the screen to the start of the rTMS pulse train. Every mapping session as well as the baseline was video-recorded for objective *post hoc* analysis (Lioumis et al., 2012; Picht et al., 2013). Each baseline picture had to be recognized before being used for the subsequent mapping session. Local pain or discomfort (e.g., in more painful temporal brain regions or the whole convexity of the hemisphere) during the mapping procedure was evaluated via a visual analog scale (VAS) and the volunteers were asked afterwards to rate the pain from 0 (no pain) to 10 points (maximal imaginable pain) (Table 1).

#### Stimulated points

**Figure 1:**
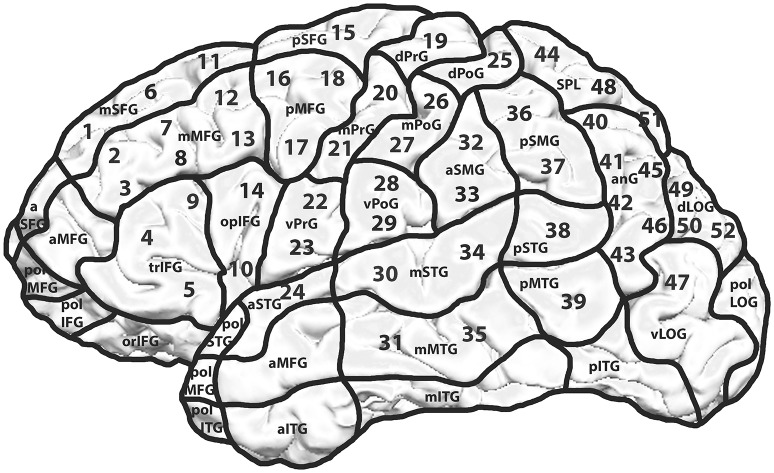
**Mapping template including the cortical parcellation system (CPS) and the 52 stimulated spots**. Mapping template with 52 predetermined spots over both hemispheres. Each cortical spot was stimulated 3 times using 5 Hz/10 pulses. The hemispheres were examined in a randomized way with an interval of 2 weeks between the two mappings. The CPS and its cortical areas are presented as published by Corina et al. (2005).

Every volunteer underwent rTMS mapping of both hemispheres on 52 predetermined cortical spots which were tagged on the 3D MRI prior to all mappings. The localizations of the stimulated spots were projected on the cortical parcellation system (CPS) published by Corina et al. (2005; Figure 1). Some CPS regions contained multiple stimulation points, for instance the angular gyrus (AnG) with 6 spots in total. Every spot was stimulated three times.

### Data analysis

Each video of the recorded rTMS sessions was analyzed as described in previous publications (Picht et al., 2013; Sollmann et al., 2013; Krieg et al., 2014b). In any given case, the investigator was blinded to all stimulated cortical spots as well as to all previous results. Any impairment of face processing or language performance was compared to the baseline, and the rTMS-induced errors were categorized into the following error types:

- error distribution for all error types
- anomia (no answer at all during stimulation)
- hesitation errors (delayed answer during stimulation)
- “similar person errors”
- “wrong person errors”

A named person was considered similar when the outward appearance was similar to the appearance of the displayed person. For example, Marilyn Monroe was displayed but Madonna was the given answer to this picture. In cases of completely different outward appearance, such as different gender, the answer was noted as wrong person. An example of this would be displaying the picture of Marilyn Monroe while the subject is naming it Bud Spencer. If at least one out of the three stimulations per spot evoked any error, the associated cortical region was considered positive for an error for the facial processing task.

The error rates (ER) were evaluated in the following ways:

1. ER for all errors per total number of stimulations; therefore, the ER describes the actual number of mistakes or errors per specified category, expressed as a percentage.
2. Number of subjects who generated errors per all stimulated subjects.

Moreover, the individual stimulation points were assigned to different lobes (frontal: spot = SP 1–23; parietal: SP 25–29, 32, 33, 36, 37, 40–46, 48, 51; occipital: SP 49, 50, 52; temporal: SP 24, 30, 31, 34, 35, 38, 39, 47).

### Statistical analysis

By the use of a Chi-squared test, all generated errors of all stimulations within the right vs. the left hemisphere were compared. We also tested the differences in ER between the two hemispheres using the Mann-Whitney-Wilcoxon test for nonparametric distribution. In this case we compared the ER for all errors of all subjects, separated for each task (GraphPad Prism 6.04, La Jolla, CA, USA). The level of significance was 0.05 (two-sided) for every statistical test. The ER was defined as the quotient of the number of rTMS-induced face processing errors divided by the number of facial processing tasks, rTMS pulses, or number of subjects.

## Results

### Performance during rTMS mapping

The mapping was tolerated well by all volunteers. Table 1 provides an overview of subject and mapping characteristics.

### Error rate relative to all stimulations

#### Error distribution for all error types

Table 2 gives an overview of the different error types. We generated the highest ER (42%) in the right middle middle frontal gyrus (mMFG) (SP 8) as well as in the left hemisphere's triangular inferior frontal gyrus (trIFG) (SP 4; ER 30%; Tables 2, 3). The overall ER of both hemispheres was 18%, respectively. There was no statistically significant difference in the ER between both hemispheres (*p* = 0.34).

**Table 2:** **Different errors per stimulation point**.

**Stimulation spot**	**Anomia**		**Hesitation**		**Similar person**		**Wrong person**		**All errors**	
	**Errors**	**Ratio**	**Errors**	**Ratio**	**Errors**	**Ratio**	**Errors**	**Ratio**	**Errors**	**Ratio**
**(a) LEFT HEMISPHERE**										
1	2	0.03	7	0.12	0	0.00	0	0.00	9	0.15
2	3	0.05	8	0.13	0	0.00	0	0.00	11	0.18
3	3	0.05	7	0.12	2	0.03	0	0.00	12	0.20
4	9	0.15	8	0.13	1	0.02	0	0.00	18	0.30
5	4	0.07	9	0.15	1	0.02	0	0.00	14	0.23
6	3	0.05	10	0.17	0	0.00	0	0.00	13	0.22
7	7	0.12	8	0.13	1	0.02	1	0.02	17	0.28
8	7	0.12	7	0.12	0	0.00	1	0.02	15	0.25
9	4	0.07	12	0.20	0	0.00	0	0.00	16	0.27
10	3	0.05	5	0.08	0	0.00	1	0.02	9	0.15
11	5	0.08	5	0.08	2	0.03	0	0.00	12	0.20
12	1	0.02	5	0.08	0	0.00	1	0.02	7	0.12
13	5	0.08	11	0.18	0	0.00	0	0.00	16	0.27
14	4	0.07	11	0.18	0	0.00	0	0.00	15	0.25
15	6	0.10	5	0.08	0	0.00	0	0.00	11	0.18
16	4	0.07	6	0.10	0	0.00	0	0.00	10	0.17
17	2	0.03	12	0.20	0	0.00	0	0.00	14	0.23
18	9	0.15	4	0.07	0	0.00	0	0.00	13	0.22
19	5	0.08	3	0.05	0	0.00	0	0.00	8	0.13
20	6	0.10	5	0.08	0	0.00	0	0.00	11	0.18
21	3	0.05	8	0.13	1	0.02	0	0.00	12	0.20
22	4	0.07	5	0.08	0	0.00	0	0.00	9	0.15
23	3	0.05	6	0.10	0	0.00	0	0.00	9	0.15
24	3	0.05	5	0.08	0	0.00	0	0.00	8	0.13
25	2	0.03	8	0.13	0	0.00	0	0.00	10	0.17
26	1	0.02	3	0.05	0	0.00	0	0.00	4	0.07
27	4	0.07	5	0.08	0	0.00	0	0.00	9	0.15
28	7	0.12	4	0.07	0	0.00	0	0.00	11	0.18
29	4	0.07	9	0.15	0	0.00	1	0.02	14	0.23
30	4	0.07	7	0.12	0	0.00	0	0.00	11	0.18
31	0	0.00	5	0.08	1	0.02	0	0.00	6	0.10
32	2	0.03	8	0.13	1	0.02	0	0.00	11	0.18
33	2	0.03	6	0.10	0	0.00	0	0.00	8	0.13
34	2	0.03	5	0.08	0	0.00	0	0.00	7	0.12
35	3	0.05	8	0.13	0	0.00	0	0.00	11	0.18
36	4	0.07	10	0.17	0	0.00	0	0.00	14	0.23
37	1	0.02	10	0.17	0	0.00	0	0.00	11	0.18
38	0	0.00	6	0.10	0	0.00	0	0.00	6	0.10
39	2	0.03	6	0.10	0	0.00	0	0.00	8	0.13
40	2	0.03	10	0.17	0	0.00	1	0.02	13	0.22
41	1	0.02	4	0.07	0	0.00	0	0.00	5	0.08
42	5	0.08	9	0.15	0	0.00	0	0.00	14	0.23
43	4	0.07	11	0.18	1	0.02	1	0.02	17	0.28
44	4	0.07	7	0.12	0	0.00	0	0.00	11	0.18
45	4	0.07	8	0.13	0	0.00	0	0.00	12	0.20
46	3	0.05	6	0.10	2	0.03	0	0.00	11	0.18
47	4	0.07	6	0.10	0	0.00	0	0.00	10	0.17
48	5	0.08	5	0.08	0	0.00	0	0.00	10	0.17
49	3	0.05	5	0.08	0	0.00	0	0.00	8	0.13
50	1	0.02	2	0.03	1	0.02	0	0.00	4	0.07
51	1	0.02	5	0.08	0	0.00	0	0.00	6	0.10
52	3	0.05	5	0.08	0	0.00	1	0.02	9	0.15
MEDIAN	3.0	0.05	6.0	0.10	0.0	0.00	0.0	0.00	11.0	0.18
MEAN	3.52	0.06	6.83	0.11	0.23	0.00	0.15	0.00	10.77	0.18
MIN	0.0	0.00	2.0	0.03	0.0	0.00	0.0	0.00	4.0	0.07
MAX	9.0	0.15	12.0	0.20	2.0	0.03	1.0	0.02	18.0	0.30
SD	2.0	0.03	2.4	0.04	0.56	0.01	0.36	0.01	3.33	0.06
**(b) RIGHT HEMISPHERE**										
1	3	0.05	5	0.08	1	0.02	0	0.00	9	0.15
2	3	0.05	9	0.15	1	0.02	0	0.00	13	0.22
3	2	0.03	8	0.13	0	0.00	0	0.00	10	0.17
4	0	0.00	6	0.10	0	0.00	0	0.00	6	0.10
5	7	0.12	12	0.20	0	0.00	0	0.00	19	0.32
6	4	0.07	13	0.22	1	0.02	0	0.00	18	0.30
7	6	0.10	9	0.15	1	0.02	0	0.00	16	0.27
8	6	0.10	19	0.32	0	0.00	0	0.00	25	0.42
9	8	0.13	8	0.13	0	0.00	0	0.00	16	0.27
10	3	0.05	7	0.12	0	0.00	2	0.03	12	0.20
11	5	0.08	15	0.25	0	0.00	0	0.00	20	0.33
12	2	0.03	11	0.18	0	0.00	0	0.00	13	0.22
13	3	0.05	10	0.17	0	0.00	0	0.00	13	0.22
14	2	0.03	8	0.13	1	0.02	0	0.00	11	0.18
15	3	0.05	8	0.13	0	0.00	0	0.00	11	0.18
16	4	0.07	6	0.10	1	0.01	2	0.02	13	0.22
17	5	0.08	6	0.10	0	0.00	0	0.00	11	0.18
18	3	0.05	6	0.10	0	0.00	1	0.01	10	0.17
19	4	0.07	4	0.07	0	0.00	0	0.00	8	0.13
20	2	0.03	7	0.12	0	0.00	0	0.00	9	0.15
21	2	0.03	6	0.10	0	0.00	0	0.00	8	0.13
22	3	0.05	7	0.12	0	0.00	0	0.00	10	0.17
23	3	0.05	10	0.17	1	0.02	0	0.00	14	0.23
24	3	0.05	5	0.08	0	0.00	0	0.00	8	0.13
25	2	0.03	5	0.08	0	0.00	1	0.02	8	0.13
26	6	0.10	8	0.13	0	0.00	0	0.00	14	0.23
27	0	0.00	7	0.12	1	0.02	0	0.00	8	0.13
28	2	0.03	7	0.12	0	0.00	1	0.02	10	0.17
29	0	0.00	4	0.07	0	0.00	0	0.00	4	0.07
30	3	0.05	1	0.02	0	0.00	0	0.00	4	0.07
31	2	0.03	11	0.18	0	0.00	0	0.00	13	0.22
32	1	0.02	8	0.13	0	0.00	0	0.00	9	0.15
33	3	0.05	13	0.22	0	0.00	0	0.00	16	0.27
34	2	0.03	12	0.20	0	0.00	0	0.00	14	0.23
35	0	0.00	4	0.07	0	0.00	0	0.00	4	0.07
36	2	0.03	9	0.15	0	0.00	0	0.00	11	0.18
37	1	0.02	10	0.17	1	0.02	0	0.00	12	0.20
38	2	0.03	7	0.12	0	0.00	0	0.00	9	0.15
39	1	0.02	6	0.10	0	0.00	0	0.00	7	0.12
40	3	0.05	12	0.20	0	0.00	0	0.00	15	0.25
41	1	0.02	5	0.08	0	0.00	0	0.00	6	0.10
42	3	0.05	7	0.12	1	0.02	0	0.00	11	0.18
43	1	0.02	11	0.18	0	0.00	0	0.00	12	0.20
44	0	0.00	4	0.07	0	0.00	0	0.00	4	0.07
45	1	0.02	6	0.10	0	0.00	0	0.00	7	0.12
46	3	0.05	6	0.10	0	0.00	0	0.00	9	0.15
47	2	0.03	6	0.10	0	0.00	0	0.00	8	0.13
48	3	0.05	4	0.07	0	0.00	0	0.00	7	0.12
49	1	0.02	11	0.18	0	0.00	0	0.00	12	0.20
50	0	0.00	3	0.05	0	0.00	0	0.00	3	0.05
51	2	0.03	6	0.10	1	0.01	1	0.02	10	0.17
52	3	0.05	5	0.08	0	0.00	0	0.00	8	0.13
MEDIAN	2.5	0.42	7.0	0.12	0.0	0.00	0.0	0.00	10.0	0.17
MEAN	2.62	0.04	7.75	0.13	0.21	0.00	0.15	0.00	10.73	0.18
MIN	0.0	0.00	1.0	0.02	0.0	0.00	0.0	0.00	3.0	0.05
MAX	8.0	0.13	19.0	0.32	1.0	0.02	2.0	0.03	25.0	0.42
SD	1.79	0.03	3.29	0.05	0.41	0.01	0.46	0.01	4.33	0.03

*A summary of different error types induced by rTMS pulse trains per stimulation spot, including all different error types. (a) Errors and error ratio found in the whole left hemisphere; (b) Errors and error ratio observed in the whole right hemisphere*.

**Table 3:** **Different error rates for all stimulations per cortical parcellation system (CPS) region and lobe**.

**CPS region/lobe**	**Anomia**		**Hesitation**		**Similar person**		**Wrong person**		**All errors**	
	**Mean errors**	**Mean ratio**	**Mean errors**	**Mean ratio**	**Mean errors**	**Mean ratio**	**Mean errors**	**Mean ratio**	**Mean errors**	**Mean ratio**
**(a) LEFT HEMISPHERE**										
AnG	3	0.05	8.5	0.14	0	0.00	0	0.00	12.5	0.21
aSMG	2	0.03	7	0.12	0.5	0.01	0	0.00	9.5	0.16
aSTG	3	0.05	5	0.08	0	0.00	0	0.00	8	0.13
dPOG	2	0.03	8	0.13	0	0.00	0	0.00	10	0.17
dPrG	5	0.08	3	0.05	0	0.00	0	0.00	8	0.13
vLOG	2	0.03	3.5	0.06	0.5	0.01	0	0.00	6	0.10
mMFG	4	0.07	9.5	0.16	0	0.00	0	0.00	15	0.25
mMTG	1.5	0.03	6.5	0.11	0.5	0.01	0	0.00	8.5	0.14
mPoG	2.5	0.04	4	0.07	0	0.00	0	0.00	6.5	0.11
mPrG	4.5	0.08	6.5	0.11	0.5	0.01	0	0.00	11.5	0.20
mSFG	3	0.05	7	0.12	0	0.00	0	0.00	12	0.20
mSTG	2	0.03	5.5	0.10	0	0.00	0	0.00	7.5	0.13
opIFG	4	0.07	11	0.18	0	0.00	0	0.00	15	0.25
pITG	4	0.07	6	0.10	0	0.00	0	0.00	10	0.17
pMFG	4	0.07	6	0.10	0	0.00	0	0.00	13	0.22
pMTG	3	0.05	6	0.10	1	0.02	0	0.00	11	0.18
polLOG	2	0.03	5	0.08	0	0.00	0.5	0.01	7.5	0.13
pSFG	6	0.10	5	0.08	0	0.00	0	0.00	11	0.18
pSMG	2.5	0.04	10	0.17	0	0.00	0	0.00	12.5	0.21
pSTG	0	0.00	6	0.10	0	0.00	0	0.00	6	0.10
SPL	4.5	0.08	6	0.10	0	0.00	0	0.00	10.5	0.18
trIFG	6.5	0.11	8.5	0.14	1	0.02	0	0.00	16	0.27
vPoG	5.5	0.09	6.5	0.11	0	0.00	0.5	0.01	12.5	0.21
vPrG	3.5	0.06	5.5	0.10	0	0.00	0	0.00	9	0.15
Frontal	102	0.07	167	0.12	8	0.01	4	0.00	281	0.20
Parietal	48	0.05	106	0.12	1	0.00	2	0.00	157	0.18
Occipital	8	0.03	17	0.07	1	0.00	1	0.00	27	0.11
Temporal	25	0.04	65	0.10	4	0.00	1	0.00	95	0.14
MEDIAN	3.0	0.05	6.0	0.10	0.0	0.00	0.0	0.00	10.3	0.17
MEAN	3.33	0.06	6.48	0.11	0.17	0.00	0.04	0.00	10.38	0.18
MIN	0.0	0.00	3.0	0.05	0.0	0.00	0.0	0.00	6.0	0.10
MAX	6.5	0.11	11.0	0.18	1.0	0.02	0.5	0.01	16.0	0.27
SD	1.50	0.03	1.93	0.03	0.31	0.01	0.14	0.00	2.79	0.05
**(b) RIGHT HEMISPHERE**										
AnG	2	0.03	6.5	0.11	0	0.00	0	0.00	9	0.15
aSMG	2	0.03	10.5	0.18	0	0.00	0	0.00	12.5	0.21
aSTG	3	0.05	5	0.08	0	0.00	0	0.00	8	0.13
dPOG	2	0.03	5	0.08	0	0.00	1	0.02	8	0.13
dPrG	4	0.07	4	0.07	0	0.00	0	0.00	8	0.13
vLOG	0.5	0.01	7	0.12	0	0.00	0	0.00	7.5	0.13
mMFG	3	0.05	8.5	0.14	0	0.00	0	0.00	13	0.22
mMTG	1	0.02	7.8	0.13	0	0.00	0	0.00	8.5	0.14
mPoG	3	0.05	7.8	0.13	0.5	0.01	0	0.00	11	0.18
mPrG	2	0.03	6.5	0.11	0	0.00	0	0.00	8.5	0.14
mSFG	4	0.07	13	0.22	1	0.02	0	0.00	18	0.30
mSTG	2.5	0.04	12.5	0.21	0	0.00	0	0.00	15	0.30
opIFG	3	0.05	8	0.13	0	0.0	0	0.00	12	0.20
pITG	2	0.03	6	0.10	0	0.00	0	0.00	8	0.13
pMFG	4	0.07	6	0.10	0	0.00	1	0.02	11	0.18
pMTG	1	0.02	6	0.10	0	0.00	0	0.00	9	0.15
polLOG	2.5	0.04	5.5	0.09	0.5	0.01	0.5	0.01	9	0.15
pSFG	3	0.05	8	0.13	0	0.00	0	0.00	11	0.18
pSMG	1.5	0.03	9.5	0.16	0.5	0.01	0	0.00	11.5	0.19
pSTG	2	0.03	7	0.12	0	0.00	0	0.00	9	0.15
SPL	1.5	0.03	4	0.07	0	0.00	0	0.00	5.5	0.09
trIFG	3.5	0.06	9	0.15	0	0.00	0	0.00	12.5	0.21
vPoG	1	0.02	5.5	0.09	0	0.00	0.5	0.01	7	0.12
vPrG	3	0.05	8.5	0.14	0.5	0.01	0	0.00	12	0.20
Frontal	83	0.06	200	0.14	7	0.01	5	0.00	295	0.21
Parietal	28	0.03	109	0.12	3	0.00	2	0.00	142	0.16
Occipital	6	0.03	25	0.10	1	0.00	1	0.00	33	0.14
Temporal	19	0.03	69	0.11	0	0.00	0	0.00	88	0.14
MEDIAN	2.3	0.04	7.0	0.12	0.0	0.00	0.0	0.00	9.0	0.15
MEAN	2.38	0.04	7.35	0.12	0.13	0.00	0.13	0.00	10.19	0.17
MIN	0.5	0.01	4.0	0.07	0.0	0.00	0.0	0.00	5.5	0.09
MAX	4.0	0.07	13.0	0.22	1.0	0.02	1.0	0.02	18.0	0.30
SD	1.00	0.02	2.30	0.04	0.26	0.00	0.30	0.00	2.76	0.05

*Summary of different error types regarding the error rates for all errors of all stimulations induced by the rTMS pulse trains per CPS region and lobe. (a) Errors and error ratio found in the whole left hemisphere; (b) Errors and error ratio generated in the whole right hemisphere*.

#### Anomia

The highest ER was in the right trIFG (SP 9; ER 13%). The left hemisphere showed the highest ER in the trIFG (SP 4) and in the posterior middle frontal gyrus (pMFG) (SP 18; ER 15% each; Tables 2, 3).

#### Hesitations

The highest ER was 32% in the right mMFG (SP 8). The left hemisphere's trIFG (SP 9) and pMFG (SP 17) each had an ER of 20% (Tables 2, 3).

#### Similar person

The right hemisphere generated an ER of 2% mainly in the frontal and parietal lobe. Regarding the left hemisphere, we observed the highest ER of 3% in the mMFG (SP 3), the middle superior frontal gyrus (mSFG) (SP 11), and the posterior middle temporal gyrus (pMTG) (SP 46) (Tables 2, 3).

#### Wrong person

In terms of the right hemisphere, we achieved the highest ER of 3% in the frontal lobe. Furthermore, we generated a high ER for the left hemisphere in every lobe with 2% each (Tables 2, 3).

### Error rate relative to all subjects

#### Error distribution for all error types

**Figure 2:**
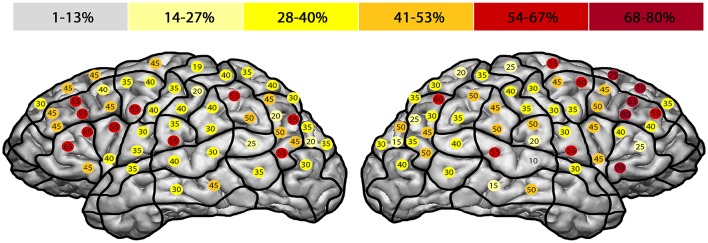
**Error rate in entire facial processing task**. This template illustrates the error rate for all errors of all subjects in the entire facial processing task. Gray represents the lowest observed error rate and dark red the highest. The highest error rates were observed in the right middle middle frontal gyrus (mMFG; stimulation point 8) with 80%, and in the left triangular inferior frontal gyrus (trIFG; stimulation points 4 & 9) with an error rate of 65% each.

**Figure 3:**
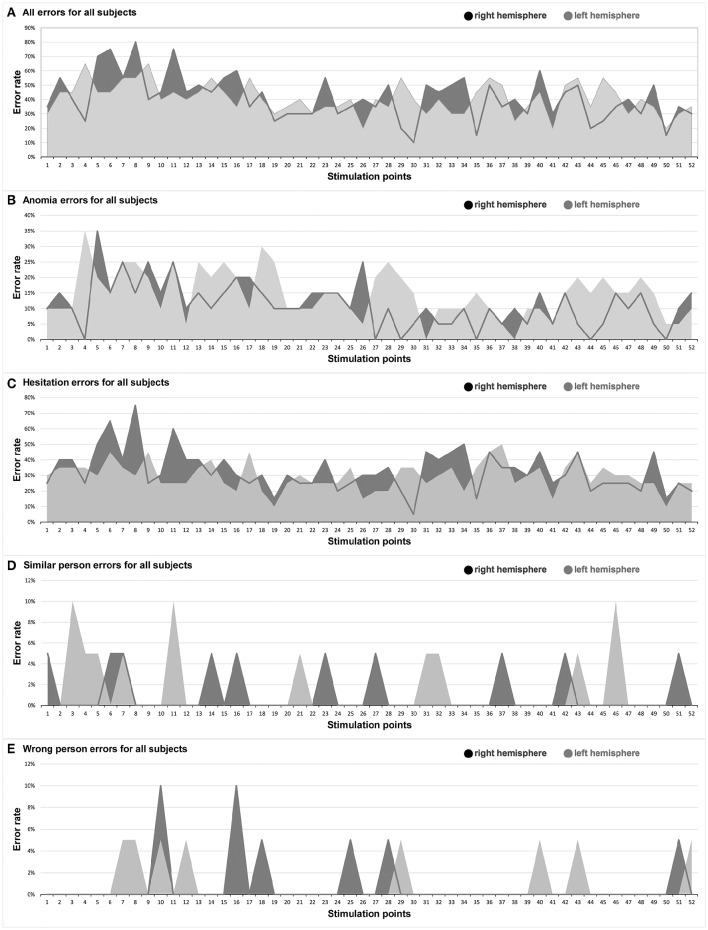
**Hemispheric comparison**. This graph illustrates the hemispheric comparison separated in each different error type in the entire facial processing mapping. Dark gray: right hemisphere, light gray: left hemisphere. **(A)** All errors for all subjects; **(B)** Anomia errors for all subjects; **(C)** Hesitation errors for all subjects; **(D)** Similar person errors for all subjects; **(E)** Wrong person errors for all subjects.

We observed the highest ER in the right mMFG (SP 8) with 80%, and in the left trIFG (SP 4 & 9), each of which had an ER of 65% (Tables 4, 5, Figures 2, 3A).

**Table 4:** **Different errors per subject**.

**Subject**	**Anomia**		**Hesitation**		**Similar person**		**Wrong person**		**All errors**	
	**Errors**	**Rate**	**Errors**	**Rate**	**Errors**	**Rate**	**Errors**	**Rate**	**Errors**	**Rate**
**(a) LEFT HEMISPHERE**										
1	2	0.01	7	0.04	2	0.01	0	0.00	11	0.07
2	8	0.05	14	0.09	1	0.01	0	0.00	23	0.15
3	29	0.19	16	0.10	0	0.00	0	0.00	45	0.29
4	3	0.19	11	0.07	1	0.01	0	0.00	15	0.10
5	10	0.06	26	0.17	0	0.00	0	0.00	36	0.23
6	7	0.04	17	0.11	1	0.01	0	0.00	25	0.16
7	10	0.06	33	0.21	3	0.02	0	0.00	46	0.30
8	6	0.04	11	0.07	0	0.00	3	0.02	20	0.13
9	16	0.10	19	0.12	0	0.00	0	0.00	35	0.22
10	10	0.06	18	0.12	2	0.01	0	0.00	30	0.20
11	16	0.10	24	0.15	1	0.01	0	0.00	41	0.26
12	32	0.21	25	0.16	0	0.00	2	0.01	59	0.38
13	0	0.00	16	0.10	0	0.00	1	0.01	17	0.11
14	4	0.03	21	0.13	0	0.00	1	0.01	26	0.17
15	7	0.04	14	0.09	0	0.00	1	0.01	22	0.14
16	5	0.03	21	0.13	0	0.00	0	0.00	26	0.17
17	2	0.01	18	0.12	1	0.01	0	0.00	21	0.13
18	3	0.02	20	0.13	0	0.00	1	0.01	24	0.15
19	3	0.02	16	0.10	1	0.01	0	0.00	20	0.13
20	10	0.06	9	0.06	0	0.00	0	0.00	19	0.12
MEDIAN	7.0	0.04	17.5	0.11	0.0	0.00	0.0	0.00	24.5	0.16
MEAN	9.15	0.06	17.8	0.11	0.65	0.00	0.45	0.00	28.05	0.18
MIN	0.0	0.00	7.0	0.00	0.0	0.00	0.0	0.00	11.0	0.07
MAX	32.0	0.21	33.0	0.21	3.0	0.02	3.0	0.02	59.0	0.38
SD	8.30	0.05	6.10	0.04	0.85	0.01	0.80	0.01	11.81	0.08
**(b) RIGHT HEMISPHERE**										
1	1	0.01	13	0.08	0	0.00	0	0.00	14	0.10
2	8	0.05	22	0.14	1	0.01	0	0.00	31	0.20
3	15	0.10	24	0.15	1	0.01	0	0.00	40	0.26
4	1	0.01	18	0.12	3	0.02	0	0.00	22	0.14
5	4	0.03	26	0.17	0	0.00	0	0.00	30	0.20
6	7	0.04	22	0.14	0	0.00	0	0.00	29	0.19
7	9	0.06	30	0.20	1	0.01	0	0.00	40	0.26
8	10	0.06	20	0.13	0	0.00	2	0.01	32	0.21
9	7	0.05	17	0.11	0	0.00	0	0.00	24	0.15
10	8	0.05	30	0.20	1	0.01	0	0.00	39	0.25
11	5	0.03	17	0.11	0	0.00	1	0.01	23	0.15
12	29	0.19	11	0.07	0	0.00	1	0.01	41	0.26
13	0	0.00	18	0.12	1	0.01	1	0.01	20	0.13
14	1	0.01	28	0.18	1	0.01	1	0.01	31	0.20
15	7	0.04	15	0.10	0	0.00	0	0.00	22	0.14
16	5	0.03	25	0.16	0	0.00	0	0.00	30	0.20
17	1	0.01	14	0.09	0	0.00	0	0.00	15	0.10
18	4	0.03	12	0.08	0	0.00	2	0.01	18	0.12
19	4	0.03	19	0.12	1	0.01	0	0.00	24	0.15
20	11	0.07	22	0.14	1	0.01	0	0.00	34	0.22
MEDIAN	6.0	0.04	19.5	0.13	0.0	0.00	0.0	0.00	29.5	0.20
MEAN	6.85	0.04	20.15	0.13	0.55	0.00	0.4	0.00	27.95	0.18
MIN	0.0	0.07	11.0	0.07	0.0	0.00	0.0	0.00	14.0	0.10
MAX	29.0	0.19	30.0	0.20	3.0	0.02	2.0	0.01	41.0	0.26
SD	6.35	0.04	5.63	0.04	0.74	0.00	0.66	0.00	8.11	0.05

*Summary of different error types induced by rTMS pulse trains per subject, including all different error types. (a) Errors and error rate observed in the whole left hemisphere; (b) Errors and error rate generated in the whole right hemisphere*.

**Table 5:** **Different error rates for all subjects per cortical parcellation system (CPS) region and lobe**.

**CPS region/lobe**	**Anomia**		**Hesitation**		**Similar person**		**Wrong person**		**All errors**	
	**Mean errors**	**Mean ratio**	**Mean errors**	**Mean ratio**	**Mean errors**	**Mean ratio**	**Mean errors**	**Mean ratio**	**Mean errors**	**Mean ratio**
**(a) LEFT HEMISPHERE**										
AnG	2.5	0.13	7	0.35	0	0.00	0	0.00	9.5	0.48
aSMG	2	0.10	6.5	0.33	0.5	0.03	0	0.00	7	0.35
aSTG	3	0.15	5	0.25	0	0.00	0	0.00	7	0.35
dPOG	2	0.10	7	0.35	0	0.00	0	0.00	8	0.40
dPrG	5	0.25	2	0.10	0	0.00	0	0.00	6	0.30
vLOG	2	0.10	3.5	0.18	0	0.00	0	0.00	5.5	0.28
mMFG	4	0.20	7	0.35	0	0.00	0	0.00	10	0.50
mMTG	1.5	0.08	6	0.30	0.5	0.03	0	0.00	7.5	0.38.
mPoG	2.5	0.13	3.5	0.18	0	0.00	0	0.00	6	0.30
mPrG	2	0.10	5.5	0.28	0.5	0.03	0	0.00	7.5	0.38
mSFG	3	0.15	6	0.30	0	0.00	0	0.00	9	0.45
mSTG	2	0.10	5.5	0.28	0	0.00	0	0.00	6	0.30
opIFG	4	0.20	8	0.40	0	0.00	0	0.00	11	0.55
pITG	3	0.25	6	0.30	0	0.00	0	0.00	6	0.30
pMFG	4	0.20	4	0.20	0	0.00	0	0.00	8	0.40
pMTG	3	0.15	6	0.30	1	0.05	0	0.00	9	0.45
polLOG	1.5	0.08	5	0.25	0	0.00	0.5	0.03	6.5	0.33
pSFG	5	0.25	5	0.25	0	0.00	0	0.00	9	0.45
pSMG	1.5	0.08	9.5	0.48	0	0.00	0	0.00	10.5	0.53
pSTG	0	0.00	5	0.25	0	0.00	0	0.00	5	0.25
SPL	3.5	0.18	5	0.25	0	0.00	0	0.00	7.5	0.38
trIFG	5.5	0.28	6.5	0.33	1	0.05	0	0.00	11	0.55
vPoG	4.5	0.23	5.5	0.28	0	0.00	0.5	0.03	9	0.45
vPrG	2.5	0.13	5	0.25	0	0.00	0	0.00	6.5	0.33
Frontal	82	0.18	139	0.30	8	0.02	4	0.01	204	0.44
Parietal	40	0.14	91	0.31	1	0.01	2	0.01	122	0.42
Occipital	7	0.09	17	0.21	0	0.00	1	0.01	24	0.30
Temporal	23	0.10	60	0.28	4	0.01	1	0.00	74	0.34
MEDIAN	2.75	0.14	5.5	0.28	0.0	0.00	0.0	0.00	7.5	0.38
MEAN	2.90	0.14	5.63	0.28	0.15	0.01	0.04	0.00	7.83	0.39
MIN	0.0	0.00	2.0	0.10	0.0	0.00	0.0	0.00	5.0	0.25
MAX	5.5	0.28	9.5	0.48	1.0	0.05	0.5	0.03	11.0	0.55
SD	1.31	0.07	1.52	0.08	0.31	0.02	0.14	0.01	1.74	0.09
**(b) RIGHT HEMISPHERE**										
AnG	2	0.10	5.5	0.28	0	0.00	0	0.00	7.5	0.38
aSMG	1	0.05	8.5	0.43	0	0.00	0	0.00	9.5	0.48
aSTG	3	0.15	4	0.20	0	0.00	0	0.00	6	0.30
dPOG	2	0.10	5	0.25	0	0.00	1	0.05	7	0.35
dPrG	2	0.10	3	0.15	0	0.00	0	0.00	5	0.25
vLOG	0.5	0.03	6	0.30	0	0.00	0	0.00	6.5	0.33
mMFG	3	0.15	8	0.40	0	0.00	0	0.00	9.5	0.48
mMTG	1	0.05	6	0.30	0	0.00	0	0.00	6.5	0.33
mPoG	2.5	0.13	6	0.30	0.5	0.03	0	0.00	7.5	0.38
mPrG	2	0.10	5.5	0.28	0	0.00	0	0.00	6	0.30
mSFG	3	0.15	12	0.60	1	0.05	0	0.00	15	0.75
mSTG	1.5	0.08	9.5	0.48	0	0.00	0	0.00	10.5	0.53
opIFG	3	0.15	6	0.30	0	0.00	0	0.00	9	0.45
pITG	2	0.10	5	0.25	0	0.00	0	0.00	8	0.40
pMFG	4	0.20	6	0.30	0	0.00	1	0.05	9	0.45
pMTG	1	0.05	6	0.30	0	0.00	0	0.00	7	0.35
polLOG	2.5	0.13	4.5	0.23	0.5	0.03	0.5	0.03	6.5	0.33
pSFG	3	0.15	8	0.40	0	0.00	0	0.00	11	0.55
pSMG	1.5	0.08	8	0.40	0.5	0.03	0	0.00	8.5	0.43
pSTG	2	0.10	7	0.35	0	0.00	0	0.00	8	0.40
SPL	1.5	0.08	4	0.20	0	0.00	0	0.00	5	0.25
trIFG	3.5	0.18	7.5	0.38	0	0.00	0	0.00	9.5	0.48
vPoG	1	0.05	5.5	0.28	0	0.00	0.5	0.03	7	0.35
vPrG	3	0.15	6.5	0.33	0.5	0.03	0	0.00	8.5	0.43
Frontal	71	0.15	169	0.37	6	0.01	5	0.01	220	0.48
Parietal	25	0.09	94	0.32	3	0.01	2	0.01	114	0.39
Occipital	6	0.08	21	0.26	1	0.01	1	0.01	26	0.33
Temporal	17	0.08	59	0.28	0	0.00	0	0.00	71	0.34
MEDIAN	2.0	0.10	6.0	0.30	0.0	0.00	0.0	0.00	7.75	0.39
MEAN	2.15	0.11	6.38	0.32	0.13	0.01	0.13	0.01	8.06	0.40
MIN	0.5	0.03	3.0	0.15	0.0	0.00	0.0	0.00	5.0	0.25
MAX	4.0	0.20	12.0	0.60	1.0	0.05	1.0	0.05	15.0	0.75
SD	0.90	0.04	0.92	0.10	0.26	0.01	0.30	0.01	2.14	0.12

*Summary of different error types concerning the error rates for all errors of all subjects induced by rTMS pulse trains per CPS region and lobe. (a) Errors and error ratio observed in the whole left hemisphere; (b) Errors and error ratio generated in the whole right hemisphere*.

#### Anomia

**Figure 4:**
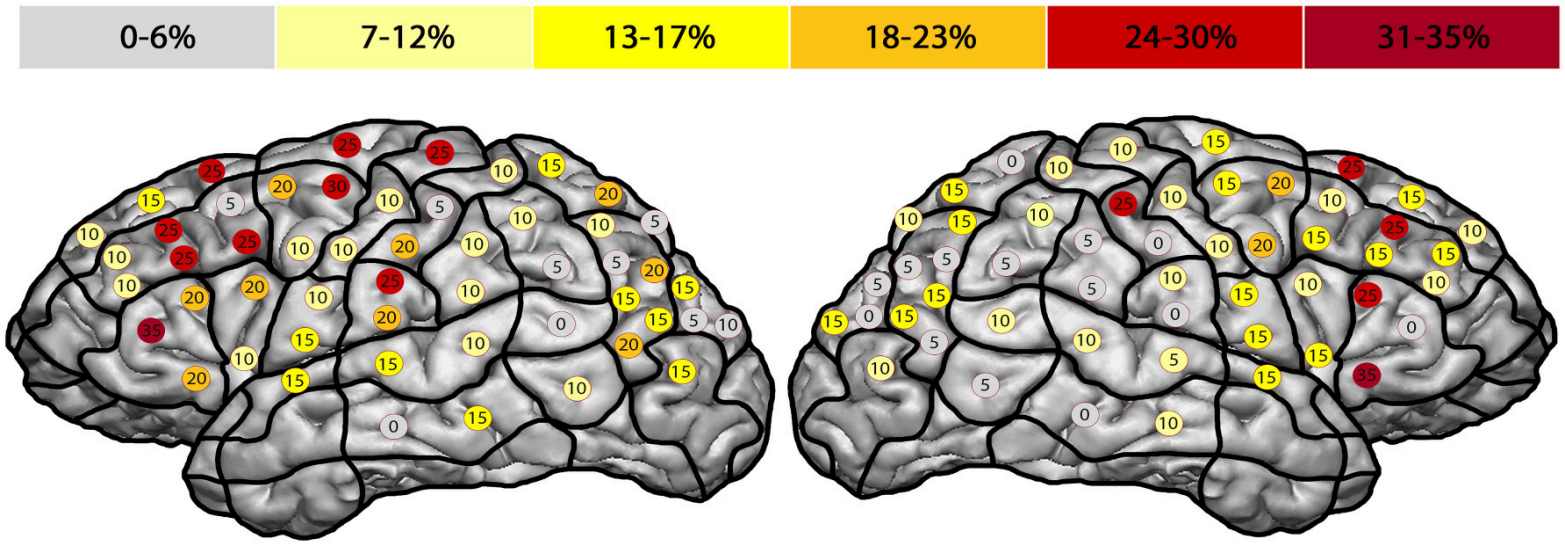
**Anomia errors**. This template shows the error rate for all anomia errors of all subjects. The highest error rates were observed in both hemispheres' triangular inferior frontal gyri, each of which had an error rate of 35% (trIFG; stimulation points 4 & 5).

The right hemisphere generated the highest ER of 35% in the trIFG (SP 5), and it was 35% in the left trIFG (SP 4) as well (Tables 4, 5, Figures 3B, 4). When comparing the left to the right hemisphere, we could not show any statistically significant difference (*p* = 0.77).

#### Hesitations

**Figure 5:**
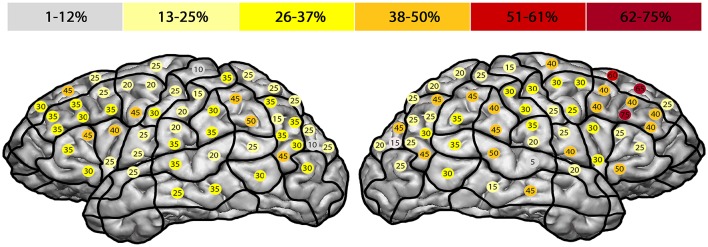
**Hesitation errors**. This template outlines the error rates for all hesitation errors in the facial processing task. The highest error rate of 75% was found in the right middle middle frontal gyrus (mMFG; stimulation point 8), followed by the left posterior supramarginal gyrus (50%) (pSMG; stimulation point 37).

The highest ER of 75% was observed in the right mMFG (SP 8). Additionally, we generated a maximum ER of 50% in the left posterior supramarginal gyrus (pSMG) (SP 37) (Tables 4, 5, Figures 3C, 5). Regarding the comparison of both hemispheres, we could not show any statistically significant difference (*p* = 0.21).

#### Similar person

The highest ER of this type was observed mainly in the right frontal lobe (10%). In the left hemisphere, we observed the highest ER (10%) in the mMFG (SP 3), mSFG (SP 11), and the pMTG (SP 46) (Tables 4, 5, Figure 3D). We could not show any statistically significant difference between both hemispheres (*p* = 0.64).

#### Wrong person

The highest ER (10%) was seen in the right opercular inferior frontal gyrus (opIFG) (SP 10) and pMFG (SP 16). The left hemisphere showed a comparable ER (5%) across spots belonging to all four lobes (Tables 4, 5, Figure 3E). Again, we did not observe any statistically significant difference between hemispheres (*p* = 0.82).

## Discussion

Facial processing has been previously examined in various studies by non-navigated TMS (Pitcher et al., 2007; Atkinson and Adolphs, 2011; Solomon-Harris et al., 2013), by fMRI (Keenan et al., 2000; Gauthier et al., 2000; Hadjikhani and De Gelder, 2002), and in several brain lesion studies (Hier et al., 1983; Young et al., 1993; Rapcsak et al., 1998; Barton, 2014; Busigny et al., 2014). Nonetheless, nTMS, with its much higher spatial resolution and therefore increased usefulness for neuroscientists, has not been used in this context before.

### Feasibility of locating cortical regions involved in facial processing

The central aim of this study is to demonstrate that rTMS is feasible for locating the specific cortical areas involved in the face processing function. Although rTMS does not allow to affect deeper brain structures, the observed superficial cortical localizations, especially in the bilateral frontal lobe, as well as in the temporal and parietal lobe, correlate with those noted in the current literature (Atkinson and Adolphs, 2011; Barton, 2014; Gomez et al., 2015; Yang et al., 2016). Furthermore, our results indicate a more important role of the right frontal lobe in visual facial processing than previously expected. Nonetheless, the exact localization of facial processing remains debatable since it involves a large variety of subfunctions and structures, including visual pathways like the OFA, memory-associated structures like the hippocampus, sensory components like the somatosensory cortex, or emotional aspects, such as those processed in the amygdala (Atkinson and Adolphs, 2011). This study provides two different facts to the scientific community: (1) rTMS could serve as an additional modality for this endeavor, maybe by serving as a seed region for diffusion tensor imaging (DTI) fiber tracking (Frey et al., 2012; Krieg et al., 2012a), and (2) since rTMS is less affected by brain tumors (Ille et al., 2015), we suggest that it could be feasible for cortical delineation of facial-processing-related areas in brain tumor patients as a stand-alone technique.

Previous studies discussed the importance of a “strict feed-forward hierarchical model of face perception” (Atkinson and Adolphs, 2011) with the OFA as the principle of a cortical network, including the FFA and the superior temporal sulcus (Dubois et al., 1999; Klopp et al., 1999; Atkinson and Adolphs, 2011; Jonas et al., 2012). However, recent neuroimaging or non-navigated TMS studies presented different findings with even more cortical areas involved in facial processing (Campanella et al., 2001; Renzi et al., 2013). Campanella et al. (2001) showed in a positron emission tomography (PET) activation study that besides the fusiform gyrus (FG), the left hemisphere's inferior frontal gyrus (IFG) and middle frontal gyrus (MFG), as well as the supramarginal gyrus (SMG) construct a facial processing network (Campanella et al., 2001). These findings are in accordance with our localizations in the frontal and parietal lobe (Figures 2–5). Renzi et al. (2013) furthermore reported on bilateral prefrontal activation for different aspects of facial processing using rTMS over the MFG and IFG (Renzi et al., 2013). In direct comparison, our study included a higher number of healthy volunteers.

When further analyzing the ER of our study, they were mostly not generated in areas that are typically associated with language, except for the left trIFG (SP 4 & 9) (Figures 2, 4; Tables 2–5). This circumstance can be regarded as further evidence for the feasibility of locating a distinctive face processing function via rTMS rather than an artifact of rTMS language mapping. Moreover, we differentiated precisely whether the errors were elicited because of possible language impairment or due to face processing impairment. Additionally, and to reduce the false positive errors due to language impairment, the volunteers were asked subsequently whether they felt unable to speak properly or unable to recognize faces. None of the participants said that he or she felt unable to speak, but in the interim unable to recognize and therefore name well-known faces.

### Comparison of the different error types and their localization

#### All errors for all error types

The highest ER were generated in the bilateral frontal lobes, with a considerably higher ER in the right hemisphere (Figure 2; Tables 2–5). We also detected high ER in the bilateral AnG, especially within the right side, even higher than in the adjoining OFA or superior temporal gyrus (STG). That might be another reference to the versatility of cortical face processing, and an indication that contradicts the discussed hierarchical system that includes just the OFA, the STG, and the FG (Dubois et al., 1999; Klopp et al., 1999; Atkinson and Adolphs, 2011; Jonas et al., 2012).

#### Anomia

The highest ER was found in the bilateral frontal lobe, especially in the trIFG (SP 4, 5, 9; Figures 3B, 4; Tables 2–5). But when we compare both whole hemispheres, we observed a maximum ER of 11% in the right hemisphere and a higher ER of 15% in the left hemisphere. Although the study was designed to eliminate possible language-related errors by differentiating whether the mistakes were generated due to language impairment or disability to speak, this might be related to the fact that anomia is often more associated to the language function, which is primarily located in the left hemisphere.

#### Hesitation errors

The highest ER was observed in the right hemisphere's mMFG (SP 8; Figures 3C, 5; Tables 2–5). Additionally, when comparing both hemispheres, we detected maximum ER in the right hemisphere of 33% in total, and 30% in the left hemisphere. In direct comparison to anomia errors and the question of whether these errors could have been made because of language impairment, we again observed more positive error localizations in the hemisphere that is non-dominant for language (Figure 5).

#### Similar and wrong person errors

In general, low error rates with a maximum of 10% were elicited (Figures 3D,E; Tables 2–5). Yet, subjects reported after stimulation that they often were unable to recognize and name a person and that the famous face suddenly felt unknown to them. In this case the volunteers generally did not respond to the presented picture at all. Thus, it was difficult in this mapping setting to evoke errors in terms of wrongly identified persons. Instead, many more hesitation errors and anomias were observed (Figures 4, 5).

### Differences between the two hemispheres and cortical regions

Additionally, to the commonly assumed localizations of facial processing in the occipitotemporal areas, the literature also provides more diverse cortical localizations (Haxby et al., 1994; Rapcsak et al., 2001; Renzi et al., 2013). Haxby et al. (1994) analyzed facial processing in a PET study by examining changes in regional cerebral blood flow (PET-rCBF). Besides their findings for face matching in the bilateral FG and the occipital and occipitotemporal cortex, they also identified the right prefrontal area and the IFG as localizations for facial processing (Haxby et al., 1994). These results confirm our observed localizations (Figure 2; Tables 2–5). As mentioned above, Renzi et al. (2013) were able to influence configurable processing of faces by using rTMS over the right IFG (Renzi et al., 2013). Rapcsak et al. (2001) tested face memory impairment in patients with focal frontal lobe lesions (Rapcsak et al., 2001). The patients showed face memory impairment compared with a cohort of control subjects, leading the researchers to conclude that the prefrontal cortices play an important role in face memory processing.

### Feasibility of preoperative mapping in brain tumor patients

Several studies have reported neuropsychological and cognitive impairment in terms of facial processing following brain damage or surgical resection of brain tumors (Hier et al., 1983; Rapcsak et al., 1998, 2001; Barton, 2014). Sanai et al. (2012) published a study with 119 glioma patients who underwent aggressive surgical glioma resection (Sanai et al., 2012); 8.4% of the patients suffered from new postoperative neuropsychological impairment, including prosopagnosia. Sergent and Villemure (1989) furthermore reported prosopagnosia in a right hemispherectomized female patient (Sergent and Villemure, 1989). Her other intellectual and cognitive functions were normal or just marginally impaired, but she was not able to identify familiar faces at all.

Besides the feasibility of using rTMS for neuropsychological or neuroscience research, this technique might also be useful for neurosurgeons regarding preoperative mapping of important neuropsychological functions in brain tumor patients (Maurer et al., 2016).

### Limitations

Due to the test algorithm we used, we are not able to completely determine whether the evoked errors in face processing were due to induced impairment of face processing or due to impairment of language or visual function.

Another limitation of mapping cortical neuropsychological functions is that some regions of the brain cannot be reached by rTMS.

In this context, the combination of rTMS with DTI fiber tracking or fMRI might be a promising modality, as already published for motor function (Krieg et al., 2012a; Frey et al., 2014) and language function (Ille et al., 2015).

Moreover, intraoperative validation is lacking for the mapping of neuropsychological functions but should be the next step.

## Conclusions

rTMS seems feasible for locating the cortical facial processing function and evoking prosopagnosia-like symptoms. Having the limitations of rTMS in mind, the observed localizations are in accordance with the current literature. Moreover, rTMS could serve as a useful adjunct to other established modalities.

## Author contributions

SM: manuscript preparation, data acquisition, data handling, data analysis, statistics, literature review. NT: data handling, data analysis, statistics. SK: manuscript preparation, data handling, data analysis, study supervision, statistics, literature review. All others: data handling, manuscript revision, study supervision.

## Funding

The study was completely financed by institutional grants of the Department of Neurosurgery and the Section of Neuroradiology, Technische Universität München.

### Conflict of interest statement

SK and FR are consultants for BrainLAB AG (Feldkirchen, Germany). SK is a consultant for Nexstim Plc (Helsinki, Finland). The authors declare that the research was conducted in the absence of any commercial or financial relationships that could be construed as a potential conflict of interest. The study was completely financed by institutional grants of the Department of Neurosurgery and the Section of Neuroradiology, Technische Universität München.

## Abbreviations

aMTG: anterior middle temporal gyrus
AnG: angular gyrus
CI: confidence interval
CPS: cortical parcellation system
DCS: direct cortical stimulation
DTI: diffusion tensor imaging
ER: error rate
FFA: fusiform face area
FG: fusiform gyrus
fMRI: functional magnetic resonance imaging
IFG: inferior frontal gyrus
IPI: inter-picture interval
ITG: inferior temporal gyrus
MFG: middle frontal gyrus
mMFG: middle middle frontal gyrus
mPoG: middle postcentral gyrus
MRI: magnetic resonance imaging
mSFG: middle superior frontal gyrus
MTG: middle temporal gyrus
nTMS: navigated transcranial magnetic stimulation
OFA: occipital face area
opIFG: opercular inferior frontal gyrus
orIFG: orbital part of the inferior frontal gyrus
PET: positron emission tomography
pMFG: posterior middle frontal gyrus
pMTG: posterior middle temporal gyrus
polLOG: polar lateral occipital gyrus
polSFG: polar superior frontal gyrus
polSTG: polar superior temporal gyrus
pSMG: posterior supramarginal gyrus
PTI: picture-to-trigger interval
TMS: transcranial magnetic stimulation
trIFG: triangular inferior frontal gyrus
rMT: resting motor threshold
rTMS: repetitive navigated transcranial magnetic stimulation
SD: standard deviation
SP: stimulation point
SFG: superior frontal gyrus
SMG: supramarginal gyrus
STG: superior temporal gyrus
vPrG: ventral precentral gyrus
VAS: visual analog scale.
