# Supplementary material for: Non-invasive Mapping of Face Processing by Navigated Transcranial Magnetic Stimulation
Source: Front Hum Neurosci. 2017 Jan 23;11:4. doi: 10.3389/fnhum.2017.00004 (PMC5253359; doi:10.3389/fnhum.2017.00004)
Supplement: Supplementary file 1 [file DataSheet1.docx]

**Supplemental Data**

Methods

rTMS Mapping

Experimental Setup

The nTMS device included a magnetic stimulator with a biphasic figure-of-eight TMS coil with a radius of 50 mm as reported earlier (Krieg et al., 2013;Picht et al., 2013;Tarapore et al., 2013). The stimulator was connected to an infrared tracking system (Polaris Spectra, Waterloo, Ontario, Canada) (Ilmoniemi et al., 1999;Ruohonen and Karhu, 2010). The T1-weighted MRI of each participant was referenced anatomically to visualize the specific brain area receiving rTMS pulses by a stereotactic camera to identify the coil position (Ilmoniemi et al., 1999;Ruohonen and Ilmoniemi, 1999;Ruohonen and Karhu, 2010). Initially, the rMT was defined by motor mapping of the cortical hand area of both hemispheres (musculus abductor pollicis brevis and/or musculus abductor digiti minimi), as reported in previous studies (Krieg et al., 2012). Mapping was then performed using 100% rMT. The rTMS mapping was performed with a frequency of 5 Hz and 10 pulses (2 s) per set of stimuli for each train. Every mapping was performed by the first author, who underwent rTMS training on healthy subjects as well as manufacturer certification prior to this study to exclude learning curve effects.

Facial Processing Task

After finishing the baseline assessment, every volunteer obtained an individual amount of various pictures, depending on the general knowledge concerning popular persons. In some cases, the subjects were mapped with just 19 correctly named pictures, while others were examined with 62 correctly identified pictures out of the baseline set of pictures. During the mapping the correctly identified pictures were presented again in a randomized and continuously repeated way. After two weeks, the subjects had to undergo the baseline assessment consisting of the same 80 pictures again to create a new and updated baseline for the second mapping of the other hemisphere.

References

Ilmoniemi, R.J., Ruohonen, J., and Karhu, J. (1999). Transcranial magnetic stimulation--a new tool for functional imaging of the brain. *Crit Rev.Biomed.Eng* 27**,** 241-284.

Krieg, S.M., Shiban, E., Buchmann, N., Gempt, J., Foerschler, A., Meyer, B., and Ringel, F. (2012). Utility of presurgical navigated transcranial magnetic brain stimulation for the resection of tumors in eloquent motor areas. *J Neurosurg* 116**,** 994-1001.

Krieg, S.M., Sollmann, N., Hauck, T., Ille, S., Foerschler, A., Meyer, B., and Ringel, F. (2013). Functional language shift to the right hemisphere in patients with language-eloquent brain tumors. *PLoS One* 8**,** e75403.

Picht, T., Krieg, S.M., Sollmann, N., Rosler, J., Niraula, B., Neuvonen, T., Savolainen, P., Lioumis, P., Makela, J.P., Deletis, V., Meyer, B., Vajkoczy, P., and Ringel, F. (2013). A comparison of language mapping by preoperative navigated transcranial magnetic stimulation and direct cortical stimulation during awake surgery. *Neurosurgery* 72**,** 808-819.

Ruohonen, J., and Ilmoniemi, R.J. (1999). Modeling of the stimulating field generation in TMS. *Electroencephalogr.Clin.Neurophysiol.Suppl* 51**,** 30-40.

Ruohonen, J., and Karhu, J. (2010). Navigated transcranial magnetic stimulation. *Neurophysiol.Clin.* 40**,** 7-17.

Tarapore, P.E., Findlay, A.M., Honma, S.M., Mizuiri, D., Houde, J.F., Berger, M.S., and Nagarajan, S.S. (2013). Language mapping with navigated repetitive TMS: Proof of technique and validation. *Neuroimage* 82**,** 260-272.
